# Evaluation of changes in the clinical benefits of oncology drugs over time following reimbursement using the ASCO-VF and the ESMO-MCBS

**DOI:** 10.1007/s00432-023-05587-0

**Published:** 2024-03-04

**Authors:** Na Ri Yoon, Young Jin Na, Jong Hwan Lee, Inmyung Song, Eui-Kyung Lee, Mi-Hai Park

**Affiliations:** 1 School of Pharmacy, Sungkyunkwan University, 2066 Seobu-ro, Jangan-gu, Suwon-si, Gyeonggi-do Republic of Korea; 2 College of Nursing and Health, Kongju National University, 56 Gongjudaehak-ro, Gongju, Republic of Korea

**Keywords:** Clinical benefit, Oncology drug, Reimbursement, ASCO-VF, ESMO-MCBS

## Abstract

**Purpose:**

This study aims to estimate changes in the value of oncology drugs over time from initial data of the reimbursement decisions to subsequent publications in Korea, using two value frameworks.

**Methods:**

We retrieved primary publications assessed for reimbursement between 2007 and July 2021 from the decision documents of Health Insurance Review and Assessment and subsequent publications made available following reimbursement decision from ClinicalTrials.Gov and PubMed databases. Changes in the clinical benefit scores were assessed using the American Society of Clinical Oncology Value Framework (ASCO-VF) and the European Society for Medical Oncology Magnitude of Clinical Benefit Scale (ESMO-MCBS). A paired *t* test was performed to test whether there was a difference in the scores between primary and subsequent publications.

**Results:**

Of 73 anticancer product/indication pairs, 45 (61.6%) had subsequent publications, of which 62.5% were released within 1 year of reimbursement decision. The mean ESMO-MCBS and ASCO-VF Net Health Benefit scores increased from primary to subsequent publications, although the differences were not significant. The mean ASCO-VF bonus score significantly increased from 15.91 to 19.09 (*p* = 0.05). The ESMO-MCBS and bonus scores increased by 0.25 and 0.21, respectively, and the bonus score had a greater impact on the ESMO-MCBS score than the preliminary score did.

**Conclusion:**

The value of drugs demonstrated in subsequent publications varies considerably among oncology drugs, depending on uncertainty associated with the initial evidence and the availability of updated evidence. As decision-making in the face of uncertainty becomes more prevalent, the value frameworks can serve as simple screening tools for re-evaluation in these cases.

**Supplementary Information:**

The online version contains supplementary material available at 10.1007/s00432-023-05587-0.

## Background

There has been increasing development of anticancer drugs and growing demand for early access to these drugs, especially among patients who lack adequate therapies (Booth and Detsky 2019). This has led to a rise in the approval of these drugs, often based on the results of single-arm and phase 2 clinical trials (Kim and Prasad 2015; Prasad et al. 2015). However, basing reimbursement decisions on data from early-phase trials or interim data increases uncertainties (Raymakers et al. 2020). If a drug is granted conditional marketing approval, the drug faces either non-reimbursement or conditional reimbursement decision (Andersen et al. 2019). Even emerging evidence after reimbursement decision adds to the uncertainties, especially due to the escalating costs of oncology drugs (Mariotto et al. 2011; Howard et al. 2015; Saluja et al. 2018). This is because the economic evaluation of an oncology drug is based not only on efficacy, quality of life (QoL), and adverse events but also on the price of the drug. Under the circumstances, it has become increasingly challenging to make reimbursement and pricing decisions for anticancer drugs.

As new evidence on a drug emerges, the clinical value of the drug is likely to change over time. Considering the uncertainties surrounding novel therapies and the continuous emergence of new evidence, some researchers emphasized the need for developing a strategy to reassess the clinical benefits of anticancer drugs (Jenei et al. 2021). Besides, international societies in the pertinent field, such as the European Society for Medical Oncology (ESMO) and the American Society of Clinical Oncology (ASCO), proposed value assessment tools to evaluate the clinical benefits of anticancer drugs, which have already been utilized in several studies (Del Paggio et al. 2017; Saluja et al. 2018; Wong et al. 2020; Ha et al. 2022). The ESMO developed the Magnitude of Clinical Benefit Scale (ESMO-MCBS), which estimates the comparative clinical benefits of drugs based on survival and response outcomes, QoL, and toxicity (Cherny et al. 2015, 2017). Similarly, the ASCO’s value framework (ASCO-VF) is designed to measure the net health benefit (NHB) score, based on comparative clinical efficacy, toxicity, QoL, and palliative effects of drugs (Cherny et al. 2015, 2017).

Many countries including Korea have adopted health technology assessment (HTA) to aid reimbursement decisions. However, the process of re-evaluating the economic value of a drug based on continuously updated evidence can be time-consuming and resource-intensive. If the clinical value of a drug can be readily reassessed by incorporating new data using the ESMO-MCBS or ASCO-VF scores, these tools can serve as valuable screening tools for re-evaluation of the drug following reimbursement decision. Therefore, this study aims to estimate changes in the clinical benefits of anticancer drugs that were listed for reimbursement in Korea, based on the literature available at the time of the initial reimbursement decision (referred to herein as “primary publication”) and the paper published thereafter (“subsequent publication”). In addition, this study explores the potential for the ESMO-MCBS and ASCO-VF as screening tools for re-evaluation.

## Materials and methods

### Identification of product/indication pairs and clinical trial extraction

**Fig. 1:**
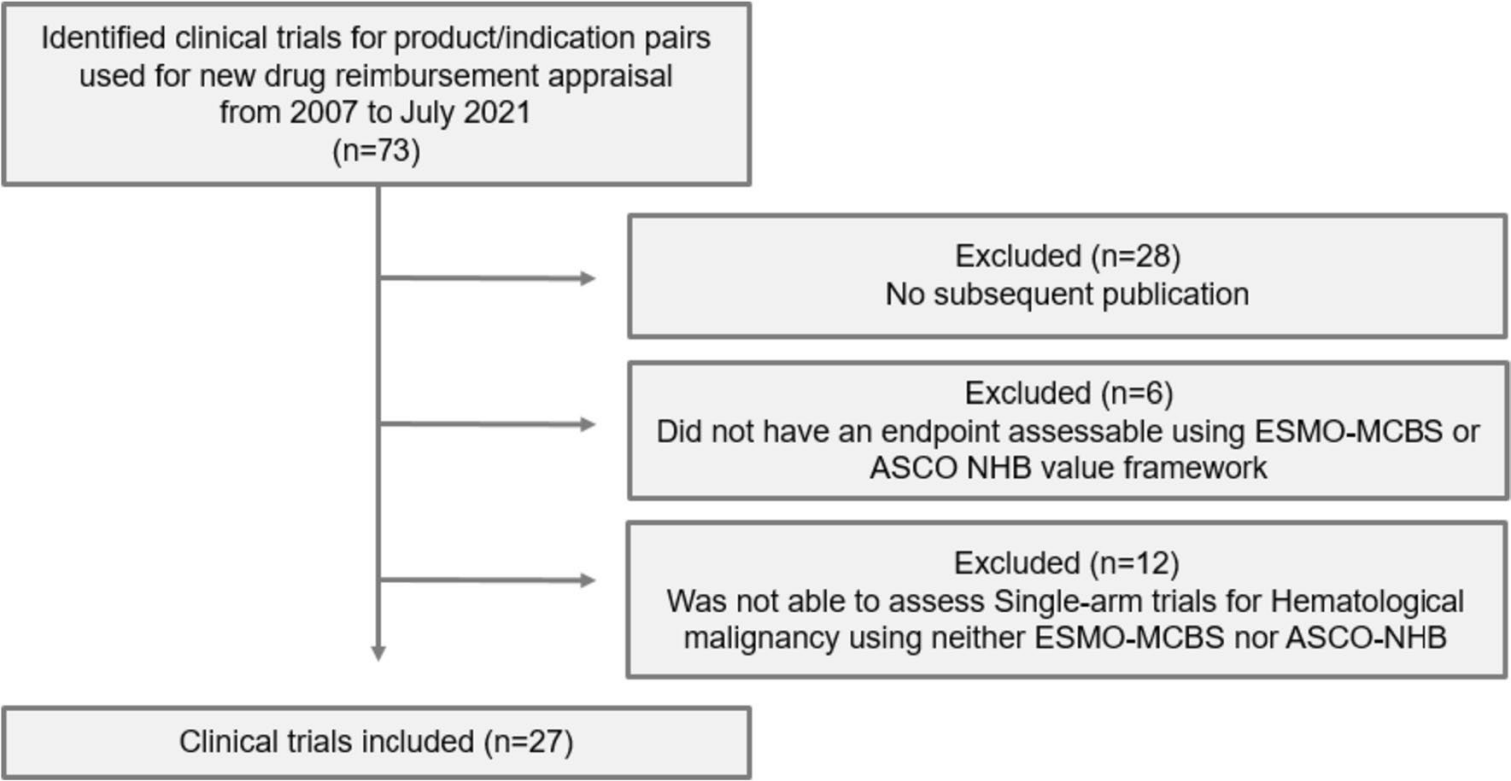
Flow diagram of the included clinical trials

From 2007 when Korea introduced the positive listing system for drug reimbursement to July 2021 when the data analysis was carried out, a total of 63 anticancer drugs have been listed for reimbursement. The 63 drugs equate to 73 “product/indication pairs” due to the nature of reimbursement appraisal. That is, for a drug with multiple indications, each indication is appraised for reimbursement. We identified 73 clinical trials for the 73 product/indication pairs to be included for analysis (Fig. 1).

We extracted primary publications for the 73 clinical trials that were considered during the initial appraisal for each indication, guided by the reports of the Health Insurance Review and Assessment. We then retrieved subsequent publications from ClinicalTrials.Gov and PubMed that have been reported after reimbursement decisions were made. A subsequent publication is defined as any report on additional results from the same trial following the initial report or on the results of a new clinical trial for the same indication. If the primary publication was based on a single-arm and phase 1 or phase 2 study, a report on a randomized phase 3 trial was regarded as a second publication.

Product/indication pairs meeting the following criteria were excluded from analysis: (1) no subsequent publication; (2) endpoints that were not assessable with the ESMO-MCBS or the ASCO-VF; and (3) single-arm trials of hematological cancer. Single-arm trials for orphan diseases with high unmet needs can only be evaluated using the ESMO-MCBS, whereas the trials for hematological cancer can be evaluated using the ASCO-VF.

### ESMO-MCBS and ASCO-VF scoring

Based on the median survival gain and the hazard ratio of the primary endpoint, the preliminary ESMO-MCBS score was calculated. The preliminary score was then downgraded or upgraded by one level, based on QoL, toxicity, and long-term data maturity, to obtain the ESMO-MCBS score. An additive or subtractive score (+ 1 or − 1) to the preliminary score was defined as the bonus score for the ESMO-MCBS (Cherny et al. 2015, 2017). Among the available ESMO-MCBS form types, form 2a (for therapies with a primary endpoint of overall survival [OS]), form 2b (for therapies with a primary endpoint of progression-free survival [PFS]), and form 3 (for single-arm studies in orphan disease and for disease with high unmet need when primary outcome is PFS or objective response rate) were used.

The ASCO-VF NHB score is the sum of clinical benefit, toxicity, and bonus scores. The clinical benefit score was calculated based on survival endpoints (OS, PFS, and response rate). The toxicity score was calculated as the proportion of patients with toxicity in the treatment arm compared with the comparator arm. The bonus score was calculated based on the tail of the curve for palliation, QoL, and treatment-free interval (Schnipper et al. 2015, 2016). Three researchers independently calculated each score. If there were discrepancies, the final scores were agreed upon through mutual discussion. Of the two types of the ASCO-VF framework (that is, one for advanced disease and the other for potentially curable [adjuvant] therapy) available, only the framework for advanced disease was used in this study.

### Assessment of changes in clinical benefit

The change in clinical benefit was calculated as the difference in the ESMO-MCBS and ASCO-VF scores between two time points (namely, “Time 1” and “Time 2”). Time 1 refers to the time of reimbursement appraisal using the primary publication. Time 2 is the time for subsequent publication that followed the initial decision. If there exist multiple subsequent publications for a drug, all were included in the analysis. If a subsequent publication reported different primary endpoint compared to a primary publication, the scoring form was changed correspondingly. If there were updates for different outcomes (i.e., OS and QoL) after the reimbursement decision, each outcome was analyzed separately. A paired *t* test was performed to test if there was a difference in clinical benefit between two time points at the significance level of 0.05.

### Subgroup analysis

For subgroup analysis, publications were classified into two groups (Group A and Group B) based on the level of evidence uncertainty at the time of reimbursement appraisal. Publications with higher levels of uncertainty meeting the following criteria were assigned to Group A:

- The clinical data source considered for appraisal was a single-arm study;
- The clinical data source considered for appraisal was either a Phase 1 or Phase 2 study;
- Crossover was permitted in the clinical trial;
- Only PFS results were reported.

All other publications were assigned to Group B. Subgroup analysis used only the ESMO-MCBS score, because the ASCO-VF score is not evaluated in single-arm studies or trials with a crossover design. The *t* test was performed to test if the degree of a change in clinical benefit differs significantly between Group A and Group B.

## Results

### Characteristics of the included trials

Among the 73 clinical trials identified, 45 (61.6%) had a subsequent publication. Of the 45 trials, 6 trials were excluded because the measured endpoints could not be evaluated with a value framework and 12 single-arm trials for hematological cancer were also excluded. In the end,we included only 27 trials for analysis **(**Fig. 1**)**. The scores and references for each product/indication pair and relevant clinical trials are summarized in Supplementary Table 1. The proportion of trials that used the randomized controlled design and were phase 3 increased from 81.5% at Time 1 to 88.9% at Time 2, so did the proportion of trials reporting OS from 74.1% to 92.6%. The number of trials reporting QoL nearly doubled from 11 to 20 **(**Table 1**)**.

**Table 1:** Characteristics of clinical trials at reimbursement (Time 1) and thereafter (Time 2)

No. of trials	Time 1 (*n* = 27)	Time 2 (*n* = 27)
Randomization		
Randomized controlled trial	22 (81.5%)	24 (88.9%)
Single-arm trial	5 (18.5%)	3 (11.1%)
Trial phase		
Phase 3	22 (81.5%)	24 (88.9%)
Phase 2	4 (14.8%)	3 (11.1%)
Phase 1	1 (3.7%)	–
Crossover allowed		
No	19 (70.4%)	17 (63.0%)
Yes	8 (29.6%)	10 (37.0%)
OS or QoL data reported		
Any OS data reported	20 (74.1%)	25 (92.6%)
Any QoL data reported	11 (40.7%)	20 (74.1%)

*OS* overall survival, *QoL* quality of life

**Fig. 2:**
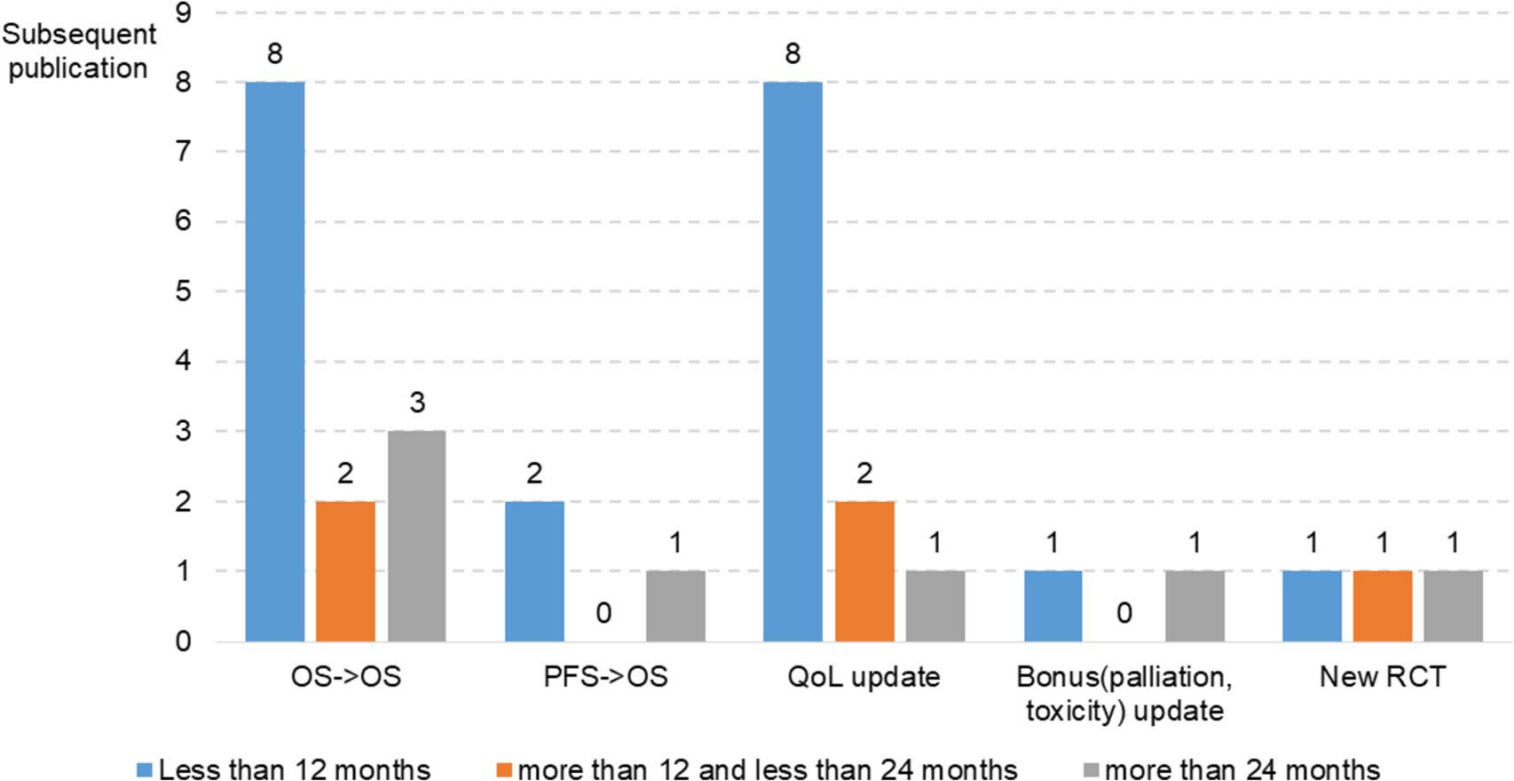
Updated evidence and time from primary publication to subsequent publication

The updated evidence covers various outcomes, including QoL, toxicity, and clinical efficacy. There was a total of 32 subsequent publications, of which 20 (62.5%) were released within 1 year of reimbursement decision (Fig. 2). Of the 20 publications, 16 had updated OS or QoL scores. Of the 16 publications regarding OS, 13 reported on long-term OS after the primary publication of OS and the remainder reported on OS after the primary publication of PFS. Additional 11 publications reported on patient-reported outcomes, such as QoL. Two and three publications reported on palliation and toxicity updates and new randomized controlled trials, respectively.

### Mean change in the ESMO-MCBS and ASCO-VF NHB score

The mean ESMO-MCBS, preliminary, and bonus scores all increased from Time 1 to Time 2, although the changes were not statistically significant. The mean difference in the ESMO-MCBS scores was + 0.25. The increase was greater for the bonus score than for the preliminary score (0.21 vs 0.04) (Table 2).

**Table 2:** Change in the mean ESMO-MCBS and ASCO-VF NHB scores

	Time 1		Time 2		Mean difference	*p* value
	Number of trials (%)	Mean score	Number of trials (%)	Mean score		
ESMO-MCBS						
ESMO-MCBS score	24 (100.0)	3.29	24 (100.0)	3.54	+ 0.25	0.137
Preliminary score	24 (100.0)	3.04	24 (100.0)	3.08	+ 0.04	0.747
Bonus score	10 (41.7)	0.25	11 (45.8)	0.46	+ 0.21	0.057
QoL upgrade	4 (16.7)	0.17	8 (33.3)	0.33		
Toxicity upgrade	6 (25.0)	0.25	7 (29.2)	0.29		
Downgrade for PFS advantage only	1 (4.2)	− 0.04	–	–		
Toxicity downgrade	1 (4.2)	− 0.04	–	–		
ASCO-VF NHB						
ASCO-VF NHB score	22 (100.0)	46.68	22 (100.0)	49.98	+ 3.30	0.081
Clinical benefit score	22 (100.0)	33.66	22 (100.0)	34.09	+ 0.43	0.621
Toxicity score	22 (100.0)	− 2.88	22 (100.0)	− 3.20	− 0.32	0.169
Bonus score	19 (86.4)	15.91	20 (90.9)	19.09	+ 3.18	0.050
OS tail of curve	4 (18.2)	3.64	3 (13.6)	2.73		
PFS tail of curve	10 (45.5)	7.27	10 (45.5)	7.27		
Palliation	6 (27.3)	2.73	11 (50.0)	5.00		
QoL	5 (22.7)	2.27	9 (40.9)	4.09		

*QoL* quality of life, *OS* overall survival, *PFS* progression-free survival

The mean ASCO-NHB, clinical benefit, and toxicity scores did not increase significantly from Time 1 to Time 2. However, the mean bonus score increased significantly from 15.91 to 19.09 (*p* = 0.05).

### Proportion (%) of trials with changes in the ESMO-MCBS and ASCO-VF NHB scores

**Fig. 3:**
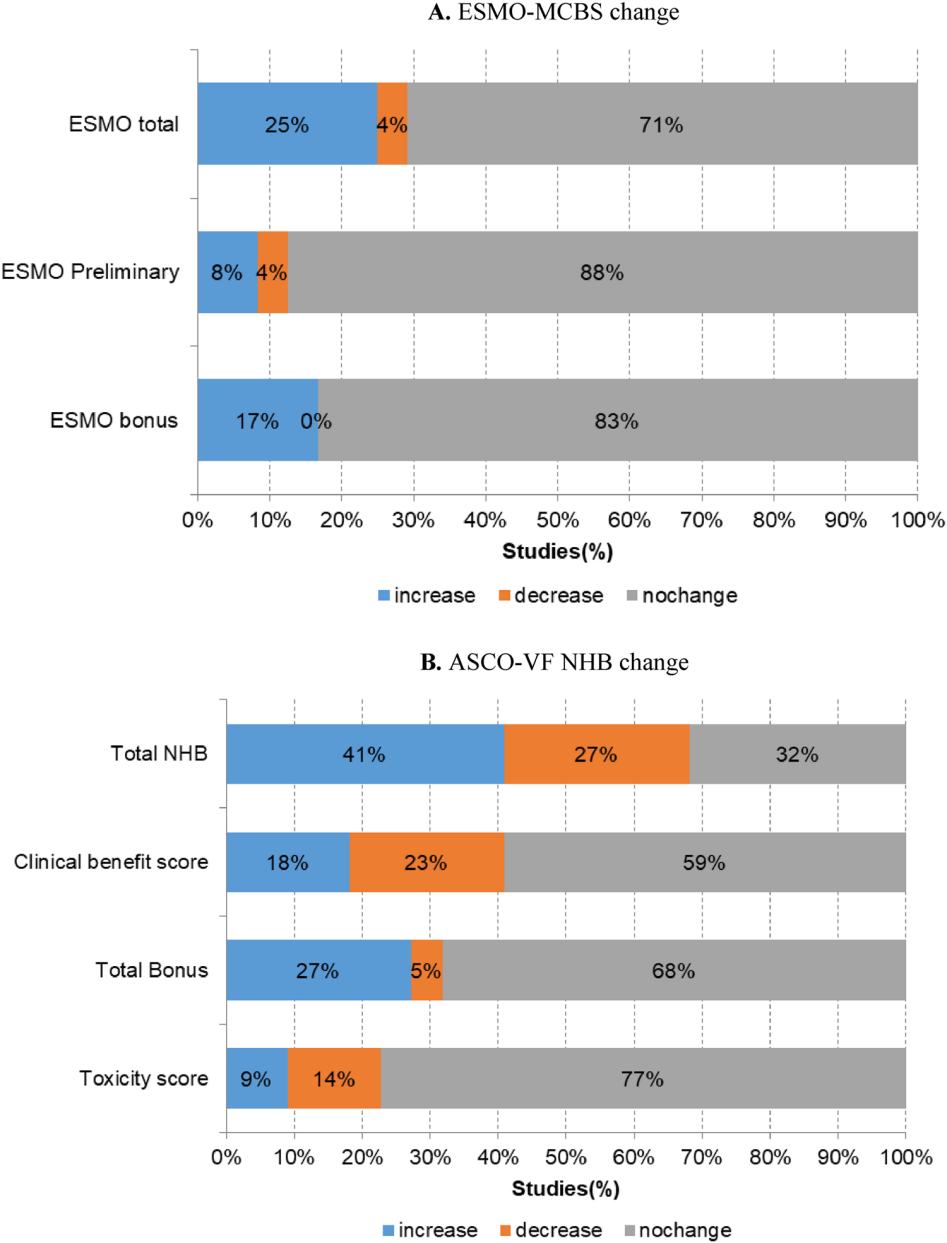
Proportion (%) of trials reporting changes in ESMO-MCBS (**A**) and ASCO-VF NHB scores (**B**)

The proportions of trials that showed changes in the total, preliminary, and bonus ESMO-MCBS scores were 29%, 12%, and 17%, respectively. The preliminary score decreased in only one trial (4%) and increased in two (8%). The remainder did not show any change. The bonus score increased in four trials (17%) (Fig. 3A). For the ASCO-VF scores, the proportions of trials with changes in the total NHB, clinical benefit, total bonus, and toxicity scores were 68%, 41%, 32%, and 23%, respectively (Fig. 3B). The clinical benefit score increased in 18% of the trials and decreased in 23%. The total bonus score increased in 27% of the trials.

### Differences in the mean ESMO-MCBS scores based on evidence uncertainty

In Group B with lower levels of evidence uncertainty, both the ESMO-MCBS and preliminary scores increased from Time 1 to Time 2. In Group A with higher levels of uncertainty, the ESMO-MCBS score increased slightly but the preliminary score decreased. The difference between Group A and Group B was not statistically significant (Table 3).

**Table 3:** Changes in the ESMO-MCBS scores between trials with different levels of uncertainty

	Mean score difference (SD)		*p* value
	Group A (*n* = 15)	Group B (*n* = 9)	
ESMO-MCBS	0.13 (0.83)	0.44 (0.73)	0.3643
Preliminary score	-0.13 (0.52)	0.33 (0.71)	0.0753
Bonus score	0.27 (0.59)	0.11 (0.33)	0.481

Group A: higher levels of evidence uncertainty; Group B: lower levels of evidence uncertainty

## Discussion

Approval and reimbursement decisions for anticancer drugs are often based on premature and insufficient evidence on clinical benefits. Over time, evidence gets updated, which changes the value of the drugs. In this current study, 61.6% of the included clinical trials updated the evidence following reimbursement appraisal. Moreover, the updates are made rather quickly. By analyzing publications issued after reimbursement decisions have already been made, we found that 62.5% of the subsequent publications were released within 1 year of reimbursement decisions. The updated evidence is shown to cover various outcomes, ranging from clinical efficacy and QoL to toxicity.

While efficacy is a crucial factor in assessing the value of a drug, this present study revealed that there were only minimal changes in average clinical efficacy from primary publication to subsequent one. The ESMO-MCBS and ASCO-VF NHB scores increased by only 0.04 and 0.43, respectively. Furthermore, the overall change in the value of anticancer drugs was substantially influenced by the bonus scores related to QoL and palliation measures.

The value of a drug demonstrated in subsequent publication varies considerably among drugs. The value increased for some drugs but decreased for others. Not only the direction but the magnitude of the change differed among drugs. Changes in the ESMO-MCBS scores ranged from − 2 to + 2, whereas changes in ASCO-MCBS scores varied more widely from − 15 to + 21.9. The value is likely to change, depending on the uncertainty associated with the initial evidence of the drug and the updated evidence.

There are discrepancies in the scoring system and factors considered between the ESMO-MCBS and the ASCO-VF NHB. For that reason, valuation results may differ for the same agent depending on the value framework used. For example, the ESMO-MCBS score for vemurafenib (therapy for melanoma) decreased from 4 at Time 1 to 2 at Time 2. This was a result of the change in the clinical benefit score according to long-term OS with crossover adjustment. On the other hand, the ASCO-VF NHB score for atezolizumab (therapy for non-small cell lung cancer) decreased from 49.7 at Time 1 to 34.7 at Time 2. This was because the tail of the curve for the bonus score was not assigned according to the long-term OS result. Despite the decrease in scores, when the other value framework was used for respective agent, changes in scores were not observed.

The proportion of clinical trials that exhibited a score change over time differed between the two frameworks used. In our analysis, only one-third of the included trials showed an ESMO-MCBS score change, which was similar to the findings of Thomson et al*.*’s (2021) study based on the European Medicine Agency’s approval data. In contrast, two-thirds (68%) of the trials showed an ASCO-VF NHB score change, which is similar to the findings of Delos Santos et al*.*’s (2021) study based on the Food and Drug Administration’s approval data. The difference between the two frameworks may have been attributable to a discrepancy in how variables were operationalized. In other words, the ESMO-MCBS scale is categorized into five levels, whereas the ASCO-VF NHB score for advanced disease is treated as a continuous variable. For that reason, the ESMO-MCBS score may have been less sensitive to changes in clinical benefit.

In our study, more than 50% of anticancer drugs were associated with high levels of uncertainty at the time of reimbursement appraisal. The mean preliminary scores for the drugs decreased after the updated evidence was made available. In some instances, a certain level of uncertainty persisted even in subsequent publications. For example, in three product/indication pairs, the primary publication was based on a non-randomized, phase 1 or 2 study, but new evidence was derived from a separate, randomized phase 3 study. However, trials following reimbursement decisions for all three cases used a crossover design, which made it difficult to measure OS gains with accuracy. In these cases, a type of uncertainty was not resolved but simply transferred into another type.

The process of conducting an economic evaluation is time-consuming and resource-intensive. The HTA of a drug for reimbursement purposes requires essential input parameters such as efficacy, QoL, and adverse events. The ASCO-VF and ESMO-MCBS tools also evaluate the value of a drug by considering multi-attribute outcomes. As decision-making in the face of uncertainty becomes more prevalent, the need for a re-evaluation process after reimbursement has also increased. As new evidence becomes available, evidence-based evaluation requires the incorporation of the updated literature. In these instances, value framework examined in this current study can serve as simple screening tools for re-evaluation. If the tools indicate a substantial decrease in the score for a drug after its reimbursement decision, there may be a need for a formal reassessment to maintain the reimbursement and pricing decisions of the drug. New evidence can either maintain, reinforce, or refute previous decisions. In South Korea, not all drugs need to be re-evaluated and the decision to re-evaluate reimbursement does not depend on the emergence of new evidence. However, for cancer drugs that are subject to the risk-sharing agreement (RSA) and exemption of economic evaluation, it is mandatory to re-evaluate reimbursement every 5 years until the contract ends.

While our study explored the potential of both frameworks as screening tools for re-evaluation after reimbursement, previous studies also investigated the relationship between the clinical benefits derived from the frameworks and reimbursement recommendations in Canada and Spain (Meyers et al. 2021; Nieto-Gómez et al. 2023). We focused on change in scores at the time of reimbursement and afterward, using both the ASCO and ESMO value frameworks. However, Meyers et al. and Nieto-Gómez et al. assessed the clinical benefit of cancer drugs that received a positive and negative recommendation for reimbursement using the ESMO-MCBS only. The previous studies showed significant differences in the ESMO-MCBS score between positive and negative decisions, but modest gains in OS. Anticancer drugs with a positive reimbursement decision had a higher proportion of RCTs and phase 3 trials, which is similar to our study. In the study by Nieto-Gómez et al., the ESMO-MCBS scores of 3 and 4 accounted for 68% of the reimbursed indications, which is comparable to the ESMO-MCBS mean score of 3.29 at the time of reimbursement observed in our study.

Despite the potential advantages of the tools, the ESMO-MCBS and ASCO-VF have several limitations that need to be considered. First, these tools do not measure clinical benefit against the best comparator used in real practice and there may be a discrepancy between the comparator considered in the reimbursement process and that used in clinical trials. Second, the ESMO-MCBS tends to focus on surrogate endpoints even though clinical benefit from surrogate endpoints may not necessarily result in final outcomes. On the other hand, the ASCO-VF tends to reflect the importance of final outcomes by attributing the greatest weight to OS. ASCO-VF cannot assess single-arm trials, which present a high level of uncertainty. For the effective application of the ESMO-MCBS and the ASCO-VF NHB to decision-making, further discussions are required as to how to reconcile discrepancies in the scoring methods and factors considered between the two tools.

Regardless of the discrepancies, the ASCO-VF and ESMO-MCBS have the potential as screening tools for re-evaluation after reimbursement decisions, which was explored in this present study. This is the first study to analyze post-reimbursement changes in value framework scores. Despite the strengths, caution should be taken in interpreting our findings due to the following limitations. First, we relied on the drugs that were listed in Korea. This choice may limit the generalizability of our findings. Nonetheless, it is worth noting that we included all anticancer drugs listed in Korea after the introduction of the positive listing system. Second, differences in the scoring methods and factors between frameworks can lead to different valuations of clinical benefits. Third, ESMO-MCBS: H-scale was published yet when the analysis was carried out in 2021. Future studies would be needed to include single-arm hematologic cancer using ESMO-MCBS:H. This limitation highlights the need to choose a framework carefully, depending on the purpose of valuation. Practical application of the framework should also consider country-specific factors, such as the reimbursement and drug pricing system and whether HTA has been implemented.

## Conclusion

Subsequent publications of oncology drugs are often released after reimbursement decisions have been made. These publications provide updated evidence on the drugs. Updated data are frequently published with a year of initial reimbursement decision. Using the ASCO-VF and ESMO-MCBS frameworks, this study revealed that while some drugs demonstrated no change in the value of clinical benefits, others exhibited notable positive or negative changes. The two value frameworks can serve as preliminary screening tools for re-evaluating agents that exhibit high uncertainty or substantial changes in scores.

## Supplementary Information

Below is the link to the electronic supplementary material.

Supplementary file1 (DOCX 177 KB)
