# Supplementary material for: Evaluation of changes in the clinical benefits of oncology drugs over time following reimbursement using the ASCO-VF and the ESMO-MCBS
Source: J Cancer Res Clin Oncol. 2024 Mar 4;150(3):113. doi: 10.1007/s00432-023-05587-0 (PMC10912263; doi:10.1007/s00432-023-05587-0)
Supplement: Supplementary file 1 — Supplementary file1 (DOCX 177 KB) [file 432_2023_5587_MOESM1_ESM.docx]

SUPPLEMENTAL MATERIAL

Evaluation of changes in the clinical benefits of oncology drugs over time following reimbursement using the American Society of Clinical Oncology Value Framework and European Society for Medical Oncology Magnitude of Clinical Benefit Scale

Supplementary Table: 1

**Supplementary Table 1.** Information of product/indication pairs and relevant trials (*n* = 27)

| **Generic name**  **(Brand name)** | **Indication** | **Study arm** | **TIME 1 Score ESMO-MCBS** | **TIME 2 score ESMO-MCBS** | **Reference(s) for ESMO scoring** | **TIME 1**  **score ASCO-VF NHB** | **TIME 2 score ASCO-VF NHB** | **Reference(s) for ASCO-VF NHB scoring** |
| --- | --- | --- | --- | --- | --- | --- | --- | --- |
| Abemaciclib  (Verzenio) | Postmenopausal Breast Cancer | Abemaciclib vs. non-steroidal aromatase inhibitor  (MONARCH 3) | 3 | 3 | [1, 2] | 28.7 | 28.7 | [1, 2] |
| Afatinib  (Giotrif) | Non-small cell lung cancer | Afatinib vs. cisplatin+pemetrexed | 4 | 4 | [3, 4] | 47.8 | 47.8 | [5] |
| Alectinib  (Alecensa) | Non-small cell lung cancer | Alectinib | 4 | 4 | [6-8] | - | - | - |
| Atezolizumab  (Tecentriq) | Non-small cell lung cancer | Atezolizumab vs. docetaxel | 5 | 5 | [9-11] | 49.7 | 34.7 | [9-11] |
| Atezolizumab  (Tecentriq) | Urothelial carcinoma | Atezolizumab | 1 | 1 | [12, 13] | - | - | - |
| Avelumab  (Bavencio) | Merkel cell carcinoma | Avelumab | 3 | 3 | [14, 15] | - | - | - |
| Brigatinib  (Alunbrig) | Non-small cell lung cancer | Brigatinib | 3 | 4 | [16, 17] | - | - | - |
| Carfilzomib  (Kyprolis) | Multiple Myeloma in combination with dexamethasone (Kd) | Carfilzomib plus dexamethasone vs. bortezomib | - | - | - | 37.1 | 59 | [18, 19] |
| Carfilzomib  (Kyprolis) | Multiple Myeloma in combination with lenalidomide plus dexamethasone | Carfilzomib, lenalidomide, and dexamethasone vs. lenalidomide plus dexamethasone | - | - | - | 29.1 | 29.3 | [20-22] |
| Ceritinib  (Zykadia) | Non-small cell lung cancer | Ceritinib vs. chemotherapy | 2 | 4 | [23, 24] | - | - | - |
| Dacomitinib  (Vizimpro) | Non-small cell lung cancer | Dacomitinib vs. gefitinib | 3 | 3 | [25-28] | 19.8 | 21.6 | [25-28] |
| Durvalumab  (Imfinzi) | Non-small cell lung cancer | Durvalumab vs. placebo | 4 | 4 | [29-31] | 41.3 | 38.3 | [29-31] |
| Enzalutamide  (Xtandi) | Prostate Cancer | Enzalutamide vs. placebo | 4 | 4 | [32-34] | 56.6 | 66.6 | [32-34] |
| Lapatinib  (Tykerb) | Breast Cancer | Lapatinib+capecitabine  vs. Capecitabine | 2 | 3 | [35-37] | 53.9 | 53.9 | [35, 37] |
| Niraparib  (Zejula) | Ovarian Cancer | Niraparib vs. placebo | 3 | 3 | [38, 39] | 76.1 | 76.1 | [38, 39] |
| Nivolumab  (Opdivo) | Non-small cell lung cancer | Nivolumab vs. docetaxel | 4 | 5 | [40-42] | 47.7 | 55.7 | [40-42]. |
| Nivolumab  (Opdivo) | Small cell lung cancer | Nivolumab vs. docetaxel | 5 | 5 | [41, 43] | 72.6 | 69.6 | [41, 43] |
| Osimertinib  (Tagrisso) | Non-small cell lung cancer | Osimertinib vs. platinum+pemetrexed | 4 | 4 | [44, 45] | 57 | 77 | [44, 45] |
| Palbociclib  (Ibrance) | Breast Cancer | Palbociclib+letrozole vs. letrozole | 3 | 3 | [46, 47] | 41.2 | 51.2 | [46, 47] |
| Pazopanib  (Votrient) | Renal cell carcinoma | Pazopanib vs. placebo | 3 | 3 | [48] | 48.2 | 48.2 | [48] |
| Pembrolizumab  (Keytruda) | Non-small cell lung cancer | Pembrolizumab vs. docetaxel | 3 | 5 | [49-51] | 45.7 | 53.5 | [49-51] |
| Pertuzumab  (Perjeta) | Breast Cancer | Pertuzumab+trastuzumab+docetaxel  vs. trastuzumab+docetaxel | 4 | 4 | [52-54] | 35.1 | 35.1 | [52, 54] |
| Ramucirumab  (Cyramza) | Gastric or gastro-esophageal junction adenocarcinoma | Ramucirumab plus paclitaxel vs placebo plus paclitaxel (RAINBOW) | 2 | 2 | [55-57] | 29.0 | 29.0 | [55-57] |
| Ribociclib  (Kisqali) | Postmenopausal Breast Cancer in combination with fulvestrant | Ribociclib+fulvestrant vs. fulvestrant alone fulvestrant MONALEESA-3 | 3 | 4 | [58] | 33.3 | 32.7 | [59] |
| Trametinib  (Meqsel) | Melanoma | Dabrafenib+trametinib | - | - | - | 72.4 | 89.2 | [60-62] |
| Trastuzumab emtansine  (Kadcyla) | Breast Cancer | Trastuzumab emtansine vs. capecitabine+lapatinib | 3 | 3 | [63-65] | 54.1 | 51.7 | [63, 65] |
| Vemurafenib  (Zelboraf) | Melanoma | Vemurafenib vs. placebo (BRIM-3) | 4 | 2 | [66, 67] | 50.6 | 50.6 | [66] |

1. Johnston, S., et al., *MONARCH 3 final PFS: a randomized study of abemaciclib as initial therapy for advanced breast cancer.* NPJ Breast Cancer, 2019. **5**: p. 5.

2. Goetz, M.P., et al., *Health-Related Quality of Life in MONARCH 3: Abemaciclib plus an Aromatase Inhibitor as Initial Therapy in HR+, HER2- Advanced Breast Cancer.* Oncologist, 2020. **25**(9): p. e1346-e1354.

3. Sequist, L.V., et al., *Phase III study of afatinib or cisplatin plus pemetrexed in patients with metastatic lung adenocarcinoma with EGFR mutations.* J Clin Oncol, 2013. **31**(27): p. 3327-34.

4. Yang, J.C., et al., *Symptom control and quality of life in LUX-Lung 3: a phase III study of afatinib or cisplatin/pemetrexed in patients with advanced lung adenocarcinoma with EGFR mutations.* J Clin Oncol, 2013. **31**(27): p. 3342-50.

5. Yang, J.C., et al., *Afatinib versus cisplatin-based chemotherapy for EGFR mutation-positive lung adenocarcinoma (LUX-Lung 3 and LUX-Lung 6): analysis of overall survival data from two randomised, phase 3 trials.* Lancet Oncol, 2015. **16**(2): p. 141-51.

6. Shaw, A.T., et al., *Alectinib in ALK-positive, crizotinib-resistant, non-small-cell lung cancer: a single-group, multicentre, phase 2 trial.* Lancet Oncol, 2016. **17**(2): p. 234-242.

7. Ou, S.H., et al., *Alectinib in Crizotinib-Refractory ALK-Rearranged Non-Small-Cell Lung Cancer: A Phase II Global Study.* J Clin Oncol, 2016. **34**(7): p. 661-8.

8. Novello, S., et al., *Alectinib versus chemotherapy in crizotinib-pretreated anaplastic lymphoma kinase (ALK)-positive non-small-cell lung cancer: results from the phase III ALUR study.* Ann Oncol, 2018. **29**(6): p. 1409-1416.

9. Rittmeyer, A., et al., *Atezolizumab versus docetaxel in patients with previously treated non-small-cell lung cancer (OAK): a phase 3, open-label, multicentre randomised controlled trial.* Lancet, 2017. **389**(10066): p. 255-265.

10. Bordoni, R., et al., *Patient-Reported Outcomes in OAK: A Phase III Study of Atezolizumab Versus Docetaxel in Advanced Non-Small-cell Lung Cancer.* Clin Lung Cancer, 2018. **19**(5): p. 441-449.e4.

11. Mazieres, J., et al., *Atezolizumab Versus Docetaxel in Pretreated Patients With NSCLC: Final Results From the Randomized Phase 2 POPLAR and Phase 3 OAK Clinical Trials.* J Thorac Oncol, 2021. **16**(1): p. 140-150.

12. Rosenberg, J.E., et al., *Atezolizumab in patients with locally advanced and metastatic urothelial carcinoma who have progressed following treatment with platinum-based chemotherapy: a single-arm, multicentre, phase 2 trial.* Lancet, 2016. **387**(10031): p. 1909-20.

13. Galsky, M.D., et al., *Atezolizumab with or without chemotherapy in metastatic urothelial cancer (IMvigor130): a multicentre, randomised, placebo-controlled phase 3 trial.* Lancet, 2020. **395**(10236): p. 1547-1557.

14. Kaufman, H.L., et al., *Updated efficacy of avelumab in patients with previously treated metastatic Merkel cell carcinoma after ≥1 year of follow-up: JAVELIN Merkel 200, a phase 2 clinical trial.* J Immunother Cancer, 2018. **6**(1): p. 7.

15. Bharmal, M., et al., *Health-related quality of life trajectory of treatment-naive patients with Merkel cell carcinoma receiving avelumab.* Future Oncol, 2020. **16**(27): p. 2089-2099.

16. Kim, D.W., et al., *Brigatinib in Patients With Crizotinib-Refractory Anaplastic Lymphoma Kinase-Positive Non-Small-Cell Lung Cancer: A Randomized, Multicenter Phase II Trial.* J Clin Oncol, 2017. **35**(22): p. 2490-2498.

17. Kawata, A.K., et al., *Converting EORTC QLQ-C30 scores to utility scores in the brigatinib ALTA study.* J Med Econ, 2019. **22**(9): p. 924-935.

18. Dimopoulos, M.A., et al., *Carfilzomib or bortezomib in relapsed or refractory multiple myeloma (ENDEAVOR): an interim overall survival analysis of an open-label, randomised, phase 3 trial.* Lancet Oncol, 2017. **18**(10): p. 1327-1337.

19. Orlowski, R.Z., et al., *Carfilzomib-Dexamethasone Versus Bortezomib-Dexamethasone in Relapsed or Refractory Multiple Myeloma: Updated Overall Survival, Safety, and Subgroups.* Clin Lymphoma Myeloma Leuk, 2019. **19**(8): p. 522-530.e1.

20. Stewart, A.K., et al., *Carfilzomib, lenalidomide, and dexamethasone for relapsed multiple myeloma.* N Engl J Med, 2015. **372**(2): p. 142-52.

21. Stewart, A.K., et al., *Health-Related Quality-of-Life Results From the Open-Label, Randomized, Phase III ASPIRE Trial Evaluating Carfilzomib, Lenalidomide, and Dexamethasone Versus Lenalidomide and Dexamethasone in Patients With Relapsed Multiple Myeloma.* J Clin Oncol, 2016. **34**(32): p. 3921-3930.

22. Siegel, D.S., et al., *Improvement in Overall Survival With Carfilzomib, Lenalidomide, and Dexamethasone in Patients With Relapsed or Refractory Multiple Myeloma.* J Clin Oncol, 2018. **36**(8): p. 728-734.

23. Kim, D.W., et al., *Activity and safety of ceritinib in patients with ALK-rearranged non-small-cell lung cancer (ASCEND-1): updated results from the multicentre, open-label, phase 1 trial.* Lancet Oncol, 2016. **17**(4): p. 452-463.

24. Shaw, A.T., et al., *Ceritinib versus chemotherapy in patients with ALK-rearranged non-small-cell lung cancer previously given chemotherapy and crizotinib (ASCEND-5): a randomised, controlled, open-label, phase 3 trial.* Lancet Oncol, 2017. **18**(7): p. 874-886.

25. Wu, Y.L., et al., *Dacomitinib versus gefitinib as first-line treatment for patients with EGFR-mutation-positive non-small-cell lung cancer (ARCHER 1050): a randomised, open-label, phase 3 trial.* Lancet Oncol, 2017. **18**(11): p. 1454-1466.

26. Mok, T.S., et al., *Improvement in Overall Survival in a Randomized Study That Compared Dacomitinib With Gefitinib in Patients With Advanced Non-Small-Cell Lung Cancer and EGFR-Activating Mutations.* J Clin Oncol, 2018. **36**(22): p. 2244-2250.

27. Mok, T.S., et al., *Updated Overall Survival in a Randomized Study Comparing Dacomitinib with Gefitinib as First-Line Treatment in Patients with Advanced Non-Small-Cell Lung Cancer and EGFR-Activating Mutations.* Drugs, 2021. **81**(2): p. 257-266.

28. Paty, J., et al., *The patient's perspective on treatment with dacomitinib: patient-reported outcomes from the Phase III trial ARCHER 1050.* Future Oncol, 2021. **17**(7): p. 783-794.

29. Antonia, S.J., et al., *Overall Survival with Durvalumab after Chemoradiotherapy in Stage III NSCLC.* N Engl J Med, 2018. **379**(24): p. 2342-2350.

30. Hui, R., et al., *Patient-reported outcomes with durvalumab after chemoradiotherapy in stage III, unresectable non-small-cell lung cancer (PACIFIC): a randomised, controlled, phase 3 study.* Lancet Oncol, 2019. **20**(12): p. 1670-1680.

31. Faivre-Finn, C., et al., *Four-Year Survival With Durvalumab After Chemoradiotherapy in Stage III NSCLC-an Update From the PACIFIC Trial.* J Thorac Oncol, 2021. **16**(5): p. 860-867.

32. Scher, H.I., et al., *Increased survival with enzalutamide in prostate cancer after chemotherapy.* N Engl J Med, 2012. **367**(13): p. 1187-97.

33. Cella, D., et al., *Impact of enzalutamide on quality of life in men with metastatic castration-resistant prostate cancer after chemotherapy: additional analyses from the AFFIRM randomized clinical trial.* Ann Oncol, 2015. **26**(1): p. 179-185.

34. Fizazi, K., et al., *Effect of enzalutamide on time to first skeletal-related event, pain, and quality of life in men with castration-resistant prostate cancer: results from the randomised, phase 3 AFFIRM trial.* Lancet Oncol, 2014. **15**(10): p. 1147-56.

35. Cameron, D., et al., *A phase III randomized comparison of lapatinib plus capecitabine versus capecitabine alone in women with advanced breast cancer that has progressed on trastuzumab: updated efficacy and biomarker analyses.* Breast Cancer Res Treat, 2008. **112**(3): p. 533-43.

36. Cameron, D., et al., *Lapatinib plus capecitabine in women with HER-2-positive advanced breast cancer: final survival analysis of a phase III randomized trial.* Oncologist, 2010. **15**(9): p. 924-34.

37. Zhou, X., et al., *Lapatinib plus capecitabine versus capecitabine alone for HER2+ (ErbB2+) metastatic breast cancer: quality-of-life assessment.* Breast Cancer Res Treat, 2009. **117**(3): p. 577-89.

38. Mirza, M.R., et al., *Niraparib Maintenance Therapy in Platinum-Sensitive, Recurrent Ovarian Cancer.* N Engl J Med, 2016. **375**(22): p. 2154-2164.

39. Matulonis, U.A., et al., *Niraparib Maintenance Treatment Improves Time Without Symptoms or Toxicity (TWiST) Versus Routine Surveillance in Recurrent Ovarian Cancer: A TWiST Analysis of the ENGOT-OV16/NOVA Trial.* J Clin Oncol, 2019. **37**(34): p. 3183-3191.

40. Borghaei, H., et al., *Nivolumab versus Docetaxel in Advanced Nonsquamous Non-Small-Cell Lung Cancer.* N Engl J Med, 2015. **373**(17): p. 1627-39.

41. Horn, L., et al., *Nivolumab Versus Docetaxel in Previously Treated Patients With Advanced Non-Small-Cell Lung Cancer: Two-Year Outcomes From Two Randomized, Open-Label, Phase III Trials (CheckMate 017 and CheckMate 057).* J Clin Oncol, 2017. **35**(35): p. 3924-3933.

42. Reck, M., et al., *Evaluation of health-related quality of life and symptoms in patients with advanced non-squamous non-small cell lung cancer treated with nivolumab or docetaxel in CheckMate 057.* Eur J Cancer, 2018. **102**: p. 23-30.

43. Brahmer, J., et al., *Nivolumab versus Docetaxel in Advanced Squamous-Cell Non-Small-Cell Lung Cancer.* N Engl J Med, 2015. **373**(2): p. 123-35.

44. Mok, T.S., et al., *Osimertinib or Platinum-Pemetrexed in EGFR T790M-Positive Lung Cancer.* N Engl J Med, 2017. **376**(7): p. 629-640.

45. Lee, C.K., et al., *Patient-Reported Symptoms and Impact of Treatment With Osimertinib Versus Chemotherapy in Advanced Non-Small-Cell Lung Cancer: The AURA3 Trial.* J Clin Oncol, 2018. **36**(18): p. 1853-1860.

46. Finn, R.S., et al., *Palbociclib and Letrozole in Advanced Breast Cancer.* N Engl J Med, 2016. **375**(20): p. 1925-1936.

47. Rugo, H.S., et al., *Impact of palbociclib plus letrozole on patient-reported health-related quality of life: results from the PALOMA-2 trial.* Ann Oncol, 2018. **29**(4): p. 888-894.

48. Sternberg, C.N., et al., *Pazopanib in locally advanced or metastatic renal cell carcinoma: results of a randomized phase III trial.* J Clin Oncol, 2010. **28**(6): p. 1061-8.

49. Herbst, R.S., et al., *Pembrolizumab versus docetaxel for previously treated, PD-L1-positive, advanced non-small-cell lung cancer (KEYNOTE-010): a randomised controlled trial.* Lancet, 2016. **387**(10027): p. 1540-1550.

50. Herbst, R.S., et al., *Long-Term Outcomes and Retreatment Among Patients With Previously Treated, Programmed Death-Ligand 1‒Positive, Advanced Non‒Small-Cell Lung Cancer in the KEYNOTE-010 Study.* J Clin Oncol, 2020. **38**(14): p. 1580-1590.

51. Barlesi, F., et al., *Health-Related Quality of Life in KEYNOTE-010: a Phase II/III Study of Pembrolizumab Versus Docetaxel in Patients With Previously Treated Advanced, Programmed Death Ligand 1-Expressing NSCLC.* J Thorac Oncol, 2019. **14**(5): p. 793-801.

52. Swain, S.M., et al., *Pertuzumab, trastuzumab, and docetaxel in HER2-positive metastatic breast cancer.* N Engl J Med, 2015. **372**(8): p. 724-34.

53. Swain, S.M., et al., *Pertuzumab, trastuzumab, and docetaxel for HER2-positive metastatic breast cancer (CLEOPATRA): end-of-study results from a double-blind, randomised, placebo-controlled, phase 3 study.* Lancet Oncol, 2020. **21**(4): p. 519-530.

54. Cortés, J., et al., *Health-related quality-of-life assessment in CLEOPATRA, a phase III study combining pertuzumab with trastuzumab and docetaxel in metastatic breast cancer.* Ann Oncol, 2013. **24**(10): p. 2630-2635.

55. Wilke, H., et al., *Ramucirumab plus paclitaxel versus placebo plus paclitaxel in patients with previously treated advanced gastric or gastro-oesophageal junction adenocarcinoma (RAINBOW): a double-blind, randomised phase 3 trial.* Lancet Oncol, 2014. **15**(11): p. 1224-35.

56. Al-Batran, S.E., et al., *Quality-of-life and performance status results from the phase III RAINBOW study of ramucirumab plus paclitaxel versus placebo plus paclitaxel in patients with previously treated gastric or gastroesophageal junction adenocarcinoma.* Ann Oncol, 2016. **27**(4): p. 673-9.

57. Cascinu, S., et al., *Tumor Response and Symptom Palliation from RAINBOW, a Phase III Trial of Ramucirumab Plus Paclitaxel in Previously Treated Advanced Gastric Cancer.* Oncologist, 2021. **26**(3): p. e414-e424.

58. Slamon, D.J., et al., *Overall Survival with Ribociclib plus Fulvestrant in Advanced Breast Cancer.* N Engl J Med, 2020. **382**(6): p. 514-524.

59. Slamon, D.J., et al., *Ribociclib plus fulvestrant for postmenopausal women with hormone receptor-positive, human epidermal growth factor receptor 2-negative advanced breast cancer in the phase III randomized MONALEESA-3 trial: updated overall survival.* Ann Oncol, 2021. **32**(8): p. 1015-1024.

60. Robert, C., et al., *Improved overall survival in melanoma with combined dabrafenib and trametinib.* N Engl J Med, 2015. **372**(1): p. 30-9.

61. Robert, C., et al., *Five-Year Outcomes with Dabrafenib plus Trametinib in Metastatic Melanoma.* N Engl J Med, 2019. **381**(7): p. 626-636.

62. Grob, J.J., et al., *Comparison of dabrafenib and trametinib combination therapy with vemurafenib monotherapy on health-related quality of life in patients with unresectable or metastatic cutaneous BRAF Val600-mutation-positive melanoma (COMBI-v): results of a phase 3, open-label, randomised trial.* Lancet Oncol, 2015. **16**(13): p. 1389-98.

63. Verma, S., et al., *Trastuzumab emtansine for HER2-positive advanced breast cancer.* N Engl J Med, 2012. **367**(19): p. 1783-91.

64. Diéras, V., et al., *Trastuzumab emtansine versus capecitabine plus lapatinib in patients with previously treated HER2-positive advanced breast cancer (EMILIA): a descriptive analysis of final overall survival results from a randomised, open-label, phase 3 trial.* Lancet Oncol, 2017. **18**(6): p. 732-742.

65. Welslau, M., et al., *Patient-reported outcomes from EMILIA, a randomized phase 3 study of trastuzumab emtansine (T-DM1) versus capecitabine and lapatinib in human epidermal growth factor receptor 2-positive locally advanced or metastatic breast cancer.* Cancer, 2014. **120**(5): p. 642-51.

66. McArthur, G.A., et al., *Safety and efficacy of vemurafenib in BRAF(V600E) and BRAF(V600K) mutation-positive melanoma (BRIM-3): extended follow-up of a phase 3, randomised, open-label study.* Lancet Oncol, 2014. **15**(3): p. 323-32.

67. Chapman, P.B., et al., *Vemurafenib in patients with BRAFV600 mutation-positive metastatic melanoma: final overall survival results of the randomized BRIM-3 study.* Ann Oncol, 2017. **28**(10): p. 2581-2587.
